# Effect of sodium-glucose co-transporter 2 inhibitor on contrast-induced acute kidney injury and prognosis in type 2 diabetes patients undergoing percutaneous coronary intervention

**DOI:** 10.3389/fmed.2025.1552539

**Published:** 2025-03-05

**Authors:** Shicheng Yang, Huifang Hao, Xiufeng Zhai, Peng Zhang, Naikuan Fu

**Affiliations:** ^1^Department of Cardiology, Tianjin University Chest Hospital, Tianjin, China; ^2^Department of Nephrology, Tianjin TEDA Hospital, Tianjin, China; ^3^Tianjin Rehabilitation and Recuperation Center, Joint Logistics Support Force, Tianjin, China

**Keywords:** sodium-glucose co-transporter 2 inhibitor, dapagliflozin, type 2 diabetes mellitus, contrast-induced acute kidney injury, prognosis

## Abstract

**Introduction:**

Contrast-induced acute kidney injury (CIAKI) is a common and serious complication following contrast administration in patients undergoing percutaneous coronary intervention (PCI). dapagliflozin, a sodium-glucose co-transporter 2 inhibitor (SGLT2i), has demonstrated renal protective effects in various clinical settings. However, the impact of dapagliflozin on the incidence of CIAKI in patients with type 2 diabetes mellitus (T2DM) undergoing PCI is not yet fully understood.

**Objective:**

To evaluate the impact of dapagliflozin on CIAKI and long-term prognosis in T2DM patients undergoing PCI.

**Methods:**

This retrospective cohort study included T2DM patients who underwent PCI at the Department of Cardiology, Tianjin University Chest Hospital, from January 2022 to June 2023. Patients were grouped based on dapagliflozin use (dapagliflozin vs. no dapagliflozin). Renal function was assessed before PCI, 48 h, and 1 week post-PCI, measuring serum creatinine, estimated glomerular filtration rate, cystatin C, and neutrophil gelatinase-associated lipocalin. All patients were followed for at least 1 year. The primary endpoint was CIAKI incidence, with secondary endpoints including renal function changes and major adverse cardiovascular events (MACE).

**Results:**

CIAKI occurred less frequently in the dapagliflozin group compared to the control group (5.8% vs. 11.7%, *χ^2^* = 4.494, *p* = 0.033). After adjusting for confounders, dapagliflozin was an independent predictor of reduced CIAKI risk (OR = 0.365, 95% CI: 0.176–0.767, *p* = 0.008). During a median 15-month follow-up, the dapagliflozin group had a lower incidence of MACE compared to the control group (Log-rank *χ*^2^ = 6.719, *p* = 0.009). Cox regression analysis showed that dapagliflozin reduced the risk of MACE (HR = 0.484, 95% CI: 0.246–0.955, *p* = 0.036).

**Conclusion:**

Chronic administration of dapagliflozin can reduces the risk of CIAKI and improves long-term cardiovascular outcomes in T2DM patients undergoing PCI. These findings support its potential use as adjunctive therapy to mitigate kidney injury and improve prognosis in this high-risk population.

## Introduction

Contrast-induced acute kidney injury (CIAKI) is a known complication following the use of iodinated contrast agents in procedures like percutaneous coronary intervention (PCI). It is characterized by a sudden decline in renal function, typically within 48 to 72 h of contrast exposure, and is associated with increased morbidity, prolonged hospitalization, and higher mortality (1, 2). Patients with type 2 diabetes mellitus (T2DM) are particularly at risk due to factors such as pre-existing chronic kidney disease (CKD), endothelial dysfunction, and hyperglycemia, which impair renal autoregulation and exacerbate nephrotoxicity (3, 4).

To date, strategies to prevent CIAKI in patients undergoing PCI have focused on reducing contrast volume, optimizing hydration, and adjusting medications. However, no pharmacologic agents have been universally adopted, and many interventions show limited efficacy (5, 6). Recently, sodium-glucose co-transporter 2 inhibitors (SGLT2i), initially developed for managing T2DM, have gained attention for their renal protective and cardiovascular benefits (7, 8). Dapagliflozin, a widely used SGLT2i, has shown promise in slowing CKD progression, reducing heart failure risk, and improving survival in patients with diabetes and cardiovascular comorbidities (9, 10). The renoprotective effects of SGLT2i are thought to stem from mechanisms such as reduced intraglomerular pressure, inflammation, oxidative stress, and fibrosis (11, 12). These benefits, along with improved glycemic control, make SGLT2i a compelling option for patients at high risk of kidney injury.

Despite promising findings, early warnings from the U.S. Food and Drug Administration (FDA) and some studies raised concerns about an increased risk of acute kidney injury (AKI) with SGLT2i use (13, 14). These warnings have affected clinicians’ confidence in the renal safety of SGLT2i, particularly in T2DM patients undergoing contrast administration. Some experts recommend discontinuing SGLT2i before contrast exposure to reduce the risk of CIAKI. However, there is no consensus on whether SGLT2i should be stopped during the perioperative period of PCI, and the impact of dapagliflozin on CIAKI risk in T2DM patients remains unclear. This study aims to assess the effect of dapagliflozin on CIAKI incidence and its impact on the prognosis of T2DM patients undergoing PCI. The results may provide evidence to support the use of dapagliflozin in managing T2DM patients at risk for CIAKI after PCI.

## Patients and methods

**Figure 1:**
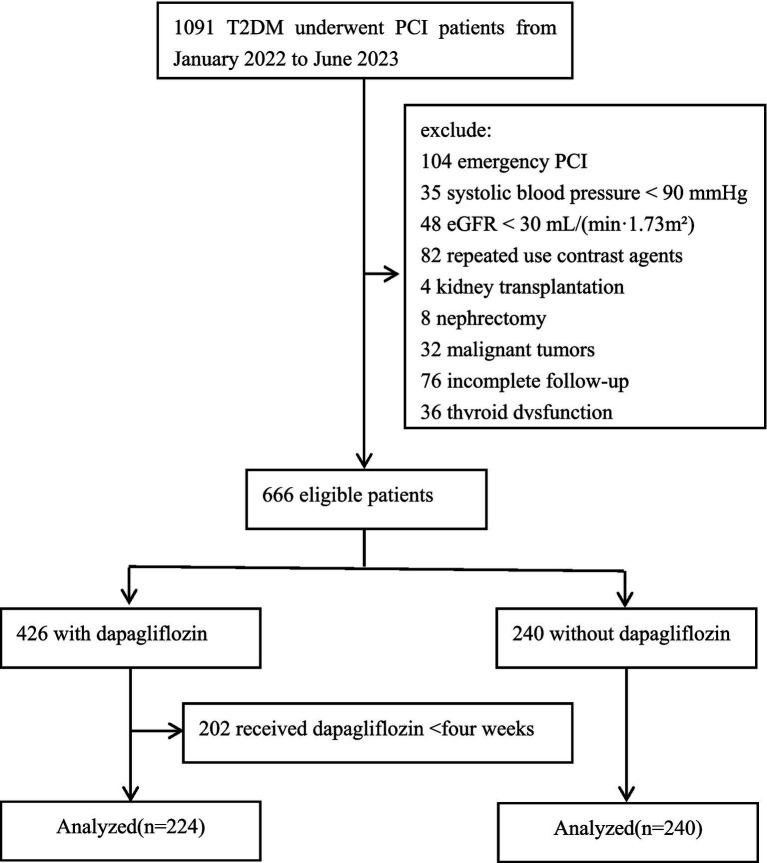
Consort flow diagram of the study. T2DM, type 2 diabetes mellitus; PCI, percutaneous coronary intervention; eGFR, estimated glomerular filtration rate.

This study adhered to the ethical guidelines of the Declaration of Helsinki. Informed consent was waived for enrolled participants, and patient confidentiality was strictly maintained. The study was approved by the Medical Ethics Committee of Tianjin University Chest Hospital (Approval no: 2023YS-1215-01). This retrospective cohort study included adult T2DM patients who underwent PCI at the Department of Cardiology, Tianjin University Chest Hospital, between January 2022 and June 2023. Patients were divided into two groups: those who received dapagliflozin and those who did not. Exclusion Criteria: (1) allergy to contrast agents or dapagliflozin, (2) emergency PCI, (3) cardiogenic shock with systolic blood pressure < 90 mmHg, (4) thyroid dysfunction, (5) estimated glomerular filtration rate (eGFR) < 30 mL/(min·1.73m^2^), (6) repeated contrast use within the past week, (7) malignant tumors, (8) previous kidney transplantation or nephrectomy, (9) incomplete follow-up. A consort flow diagram of the study is shown in Figure 1.

To minimize the risk of CIAKI, all patients received hydration therapy with sodium chloride saline (Shanghai Baxter Medical Products Co., Ltd., Batch No.: H19993745) at a rate of 1 mL/(kg·h) for 6 ~ 12 h before and after PCI. For patients with heart failure or left ventricular ejection fraction (LVEF) < 45%, the hydration rate was reduced to 0.5 mL/(kg·h). All patients were also encouraged to drink plenty of water. Enrolled patients received either iodixanol (Yangtze River Pharmaceutical Group Co., Ltd., Batch No.: H20184002), an isotonic contrast agent, or iopamidol (Jiangsu Hengrui Medicine Co., Ltd., Batch No.: H20067896), a low-osmolar contrast agent. Patients in the dapagliflozin group received 10 mg of dapagliflozin (AstraZeneca Pharmaceuticals Co., Ltd., Batch No.: H20170119) orally once daily for at least 4 weeks prior to contrast administration and continued the same dose post-PCI.

The basic information and medical history of both patient groups were recorded, including medication history, dapagliflozin duration, type and dosage of contrast agent, and hydration volume. Renal function parameters were also assessed before PCI and at 48 h and 1 week post-treatment. These included serum creatinine (Scr), eGFR, serum cystatin C (Cys-C), and neutrophil gelatinase-associated lipocalin (NGAL).

All patients were followed for at least 1 year post-PCI, with follow-up data collected via outpatient visits, hospital records, or telephone interviews. During follow-up, major adverse cardiac events (MACE)—including cardiac death, non-fatal myocardial infarction, non-fatal stroke, unplanned revascularization, and acute heart failure—were recorded. The primary endpoint was the incidence of CIAKI, defined as a Scr increase of >26.5 μmol/L (0.3 mg/dL) or > 50% from baseline within 48 h or 1 week of contrast administration, respectively (1). Secondary endpoints included changes in renal function and the occurrence of MACE during follow-up.

The eGFR was calculated using the formula: eGFR = 186 × Scr (mg/L)^−1.154^ × age (years)^−0.203^ × (female × 0.742). T2DM was defined by the presence of diabetes symptoms (e.g., polydipsia, polyphagia, polyuria, weight loss) along with any of the following blood glucose levels: postprandial glucose ≧ 11.1 mmol/L, fasting glucose ≧ 7.0 mmol/L, or glucose ≧ 11.1 mmol/L 2 h after a glucose tolerance test. Anemia was defined as hemoglobin <120 g/L or red blood cell count <4.5 × 10^12^/L for adult males, and hemoglobin <110 g/L or red blood cell count <4.0 × 10^12^/L for adult females.

### Data analysis

Descriptive statistics summarize baseline characteristics. Continuous variables were expressed as mean ± standard deviation (SD), two-way ANOVA was used for comparing multiple groups, and pairwise comparisons between groups were performed using the SNK-q test. Categorical variables were presented as frequencies and percentages. The differences between the dapagliflozin and control groups were compared using independent t-tests or Mann–Whitney U tests for continuous variables, and chi-square or Fisher’s exact tests for categorical variables. Multivariate logistic regression identified independent predictors of CIAKI, adjusting for potential confounders. The Log-rank test compared MACE occurrence between groups, while Cox regression assessed the effect of dapagliflozin on prognosis. Kaplan–Meier curves were plotted using GraphPad Prism 9.0. A *p*-value <0.05 was considered statistically significant. All statistical analyses were conducted using SPSS version 22.0 (IBM Corp., Armonk, NY, United States).

## Results

A total of 464 T2DM patients undergoing PCI were enrolled, with a mean age of 64.05 ± 15.36 years (340 males and 124 females). The dapagliflozin group included 224 patients, who had an average dapagliflozin treatment duration of 10.56 ± 2.62 weeks prior to PCI. The control group consisted of 240 patients. The dapagliflozin group had a significantly lower LVEF compared to the control group, 53.22% ± 10.24% vs. 55.46% ± 11.18% (*p* < 0.05). No other baseline characteristics showed significant differences between the groups (*p* > 0.05). This indicates that the general characteristics between the two groups of patients are comparable. As shown in Table 1. All patients completed the follow-up as scheduled.

**Table 1:** Comparison of baseline characteristics between groups.

Variable	Dapagliflozin group (*n* = 224)	Control group (*n* = 240)	*t*/*χ*^2^	*p*
Age	63.52 ± 15.70	64.18 ± 15.24	0.459	0.646
Age > 75 years	64 (28.6)	78 (32.5)	0.842	0.366
Male	161 (71.9)	179 (74.6)	0.434	0.530
Body Mass Index(kg/m^2^)	24.22 ± 3.46	24.56 ± 3.39	1.069	0.286
Duration of diabetes(years)	6.53 ± 2.77	6.32 ± 2.54	0.852	0.395
Systolic blood pressure	132.54 ± 28.72	135.48 ± 27.62	1.124	0.264
Diastolic blood pressure	82.56 ± 10.65	83.36 ± 11.03	0.794	0.428
Smoker	138 (61.6)	149 (62.1)	0.011	0.924
Alcohol consumption	106 (47.3)	118 (49.2)	0.158	0.711
Hypertension	122 (54.5)	134 (55.8)	0.088	0.780
Acute Myocardial Infarction	72 (32.1)	83 (34.6)	0.310	0.623
Stroke	39 (17.4)	44 (18.3)	0.067	0.810
Hemoglobin(g/L)	129.45 ± 30.44	128.68 ± 30.72	0.271	0.787
Red blood cell count(×10^12^)	4.71 ± 1.03	4.75 ± 1.07	0.289	0.772
Anemia	18 (8.0)	21 (8.8)	0.077	0.868
LVEF	53.22 ± 10.24	55.46 ± 11.18	2.246	0.025
LVEF<45%	99 (44.2)	84 (35.0)	4.103	0.046
eGFR<60 mL/(min^.^·1.73m^2^)	81 (36.2)	88 (36.7)	0.013	0.923
Iohexol	104 (46.4)	118 (49.2)	0.348	0.578
Iopamidol	120 (53.6)	122 (50.8)	0.348	0.578
Contrast agent dosage(mL)	189.10 ± 56.22	187.78 ± 56.54	0.252	0.801
Hydration volume(mL)	1256.57 ± 292.68	1248.42 ± 290.48	0.301	0.764
Fasting blood glucose(mmol/L)	6.12 ± 1.67	6.18 ± 1.75	0.377	0.706
Postprandial 2-h blood glucose(mmol/L)	10.88 ± 3.77	10.91 ± 3.82	0.085	0.932
Glycated hemoglobin (HbA1c)	6.28 ± 1.14	6.19 ± 1.12	0.858	0.392
Triglycerides(mmol/L)	1.96 ± 0.92	1.94 ± 0.89	0.238	0.812
Cholesterol(mmol/L)	5.68 ± 1.87	5.76 ± 1.94	0.452	0.652
High-density lipoprotein (mmol/L)	1.64 ± 0.56	1.69 ± 0.62	0.909	0.364
Low-density lipoprotein (mmol/L)	3.58 ± 1.68	3.65 ± 1.72	0.443	0.658
Statins	206(92.0)	218 (90.8)	0.188	0.742
Β-blocker	178 (79.5)	189 (78.8)	0.036	0.909
ACEI/ARB	106 (47.3)	119 (49.6)	0.237	0.643
Diuretics	58 (25.9)	69 (28.7)	0.476	0.532
Calcium channel blockers	88 (39.3)	96 (40.0)	0.025	0.924
Alpha-glucosidase inhibitors	102 (45.5)	114 (47.5)	0.180	0.710
Insulin secretagogues	78 (34.8)	86 (35.8)	0.052	0.846
Insulin	98 (43.8)	107 (44.6)	0.033	0.925
GLP-1RA	58 (25.9)	65 (27.1)	0.084	0.833

LVEF, left ventricular ejection fraction; eGFR, estimated glomerular filtration rate; ACEI/ARB, angiotensin-converting enzyme inhibitors /angiotensin II receptor blockers; GLP-1RA, glucagon-like peptide-1 receptor agonist.

Before PCI, no significant differences were found in Scr, eGFR, Cys-C, or NGAL levels between the two groups (*p* > 0.05). At 48 h post-PCI, Cys-C and NGAL levels increased in both groups compared to pre-PCI values. However, the dapagliflozin group had significantly lower Cys-C and NGAL levels than the control group (*p* < 0.05). By 1 week post-PCI, no significant differences in Scr, eGFR, Cys-C, or NGAL were observed between the two groups (*p* > 0.05), as shown in Table 2.

**Table 2:** Renal function changes pre-PCI, 48 h post-PCI, and 1 week post-PCI.

Variable	*n*	Scr (μmol·L^−1^)				
		Pre-PCI	48 h post-PCI	One week post-PCI	*F*	*p*
Dapagliflozin group	224	74.82 ± 19.67	79.12 ± 25.95	75.26 ± 24.62	1.070	0.983
Control group	240	74.71 ± 18.92	78.22 ± 26.29	74.39 ± 28.64	0.373	0.830

Variable	*n*	eGFR (mL/min·1.73m^2^)				
		Pre-PCI	48 h post-PCI	One week post-PCI	*F*	*p*
Dapagliflozin group	224	85.68 ± 30.93	83.81 ± 31.39	84.75 ± 31.54	0.506	0.605
Control group	240	85.50 ± 31.12	81.90 ± 31.54	82.63 ± 31.37	0.128	0.884

Variable	*n*	Cys-C (mg·L^−1^)				
		Pre-PCI	48 h post-PCI	One week post-PCI	F	*p*
Dapagliflozin group	224	1.04 ± 0.28	2.02 ± 0.88^ab^	1.18 ± 0.34	18.225	0.037
Control group	240	1.07 ± 0.22	2.64 ± 0.76^b^	1.17 ± 0.40	21.775	0.042

Variable	*n*	NGAL (μg·L^−1^)				
		Pre-PCI	48 h post-PCI	One week post-PCI	*F*	*p*
Dapagliflozin group	224	84.54 ± 28.42	92.23 ± 31.75^ab^	85.10 ± 29.33	41.957	0.028
Control group	240	85.36 ± 29.56	102.35 ± 35.32^b^	86.67 ± 29.76	66.951	0.018

^a^Compared to the control group (*p* < 0.05); ^b^Compared to pre-PCI (*p* < 0.05). SCR, serum creatinine; eGFR, estimated glomerular filtration rate; PCI, percutaneous coronary intervention; Cys-C, cystatin C; NGAL, neutrophil gelatinase-associated lipocalin.

A total of 41 cases of CIAKI occurred across both groups, resulting in an overall incidence of 8.8%. In the dapagliflozin group, 13 cases (5.8% incidence) were reported, while the control group had 28 cases (11.7% incidence). The difference in CIAKI incidence between the two groups was statistically significant (5.8% VS. 11.7%, *χ^2^* = 4.494, *p* = 0.033).Logistic regression analysis identified factors influencing CIAKI, including LVEF, LVEF ≤45%, eGFR <60 mL/(min·1.73m^2^), contrast agent dosage, iohexol, hydration volume, age > 75 years, anemia, and dapagliflozin. Dapagliflozin was found to be an independent protective factor against CIAKI (OR = 0.365, 95% CI: 0.176–0.767, *p =* 0.008), as shown in Table 3.

**Table 3:** Logistic regression analysis.

Variable	Univariate logistic regression analysis			Multivariate logistic regression analysis		
	*p*	OR	95% CI	*p*	OR	95% CI
LVEF	0.781	1.039	0.792 ~ 1.365	—	—	—
LVEF<45%	0.066	1.978	0.956 ~ 4.093	0.001	3.851	1.752 ~ 8.466
eGFR<60 mL/(min.1.73m^2^)	0.003	3.702	1.553 ~ 8.821	0.256	2.113	0.581 ~ 7.699
Contrast agent dosage	0.414	0.933	0.790 ~ 1.102	—	—	—
Iohexol	0.003	0.972	0.954 ~ 0.990	0.110	0.979	0.954 ~ 1.005
Hydration volume	0.637	1.000	1.000 ~ 1.001	—	—	—
Age > 75 years	0.864	1.002	0.975 ~ 1.031	—	—	—
Anemia	0.032	1.005	1.000 ~ 1.010	0.016	1.006	1.001 ~ 1.012
Dapagliflozin	0.029	0.466	0.235 ~ 0.925	0.008	0.365	0.174 ~ 0.767

LVEF, left ventricular ejection fraction; eGFR, estimated glomerular filtration rate; OR, odds ratio; CI, confidence interval.

**Figure 2:**
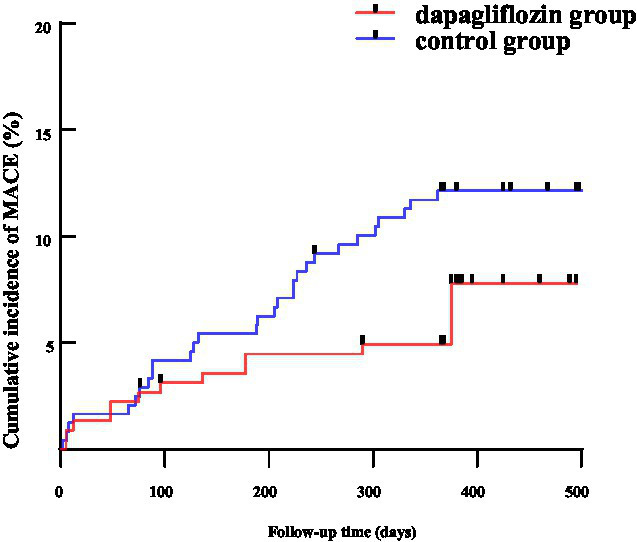
The Kaplan–Meier survival curve for dapagliflozin group and control group. MACE, major adverse cardiovascular events; CIAKI, contrast-induced acute kidney injury.

During a median follow-up of 15 months (range: 12.0–16.8 months), MACE events were recorded in both groups. In the dapagliflozin group, there were 2 cases of recurrent acute myocardial infarction, 3 cases of unplanned revascularization, 5 cases of acute heart failure, and 1 case of stroke. In the control group, 3 cases of recurrent acute myocardial infarction, 12 unplanned revascularizations, 12 acute heart failure cases, 2 strokes, and 1 sudden death occurred. Kaplan–Meier curves showed a significant benefit for dapagliflozin-treated patients (Log-rank *χ*^2^ = 6.719, *p* = 0.009), as shown in Figure 2.

**Figure 3:**
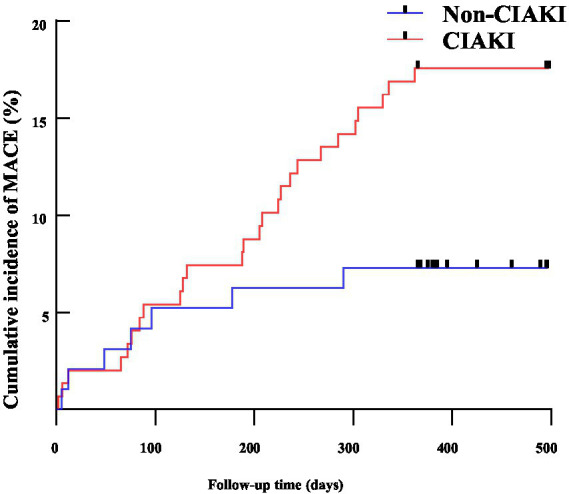
The Kaplan–Meier survival curve for CIAKI and Non CIAKI. MACE, major adverse cardiovascular events; CIAKI, contrast-induced acute kidney injury.

Among the CIAKI group, there were 2 cases of recurrent acute myocardial infarction, 1 unplanned revascularization, 3 acute heart failure cases, 1 stroke, and 1 sudden death. In the non-CIAKI group, there were 3 recurrent myocardial infarctions, 14 unplanned revascularizations, 14 acute heart failure cases, 2 strokes, and no sudden deaths. Kaplan–Meier analysis revealed that CIAKI increased the risk of MACE (Log-rank *χ*^2^ = 5.804, *p =* 0.016), as shown in Figure 3.

Cox regression analysis identified factors influencing MACE occurrence, with MACE as the dependent variable and age > 75, eGFR <60 mL/(min·1.73m^2^), LVEF ≦ 45%, anemia, AMI, CIAKI, and dapagliflozin as independent variables. The results showed that dapagliflozin improved the prognosis of T2DM patients after PCI (HR = 0.484, 95% CI: 0.246–0.955, *p* = 0.036), as shown in Table 4.

**Table 4:** COX regression analysis.

Variable	Univariate Cox regression analysis			Multivariate Cox regression analysis		
	*p*	HR	95% CI	*p*	HR	95% CI
Age > 75 years	0.035	2.214	1.057 ~ 4.638	0.584	1.280	0.529 ~ 3.098
eGFR<60(min·1.73m^2^)	0.261	1.010	0.993 ~ 1.028	–	–	–
LVEF≦45%	0.626	1.202	0.574 ~ 2.518	–	–	–
Anemia	0.028	1.121	1.013 ~ 1.242	0.271	1.062	0.954 ~ 1.183
AMI	0.935	1.001	0.969 ~ 1.035	–	–	–
CIAKI	<0.001	3.723	1.825 ~ 7.596	0.036	2.586	1.066 ~ 6.273
Dapagliflozin	0.015	0.435	0.222 ~ 0.852	0.036	0.484	0.246 ~ 0.955

eGFR, estimated glomerular filtration rate; LVEF, left ventricular ejection fraction; AMI, acute Myocardial Infarction; CIAKI, contrast-induced acute kidney injury.

## Discussion

Our study found that dapagliflozin significantly reduced the incidence of CIAKI compared to the control group. Additionally, dapagliflozin was associated with improved long-term outcomes, showing fewer MACE events during at least 1 year of follow-up. Therefore, in T2DM patients on long-term dapagliflozin, clinicians need not worry about an increased risk of CIAKI, and discontinuing dapagliflozin before contrast administration is unnecessary.

Urine output and Scr are commonly used to diagnose acute kidney injury (AKI) (15, 16), but they have limitations. Scr is influenced by factors such as weight, muscle mass, age, diet, and gender, while urine output can be affected by fluid intake, diuretics, and cardiac function, reducing their accuracy for early CIAKI diagnosis. Scr levels typically peak 2–3 days after contrast agent administration and usually return to baseline levels within 7–10 days. In contrast, Cys-C and NGAL are expressed in large amounts and released into the blood and urine within hours after the onset of AKI, significantly increasing within a short time frame (6–12 h), and then gradually decreasing. Cys-C is a more sensitive biomarker for early renal impairment, as it is less influenced by factors like muscle mass, age, and gender (17). NGAL, a protein involved in regulating apoptosis in renal tubular cells, increases rapidly in response to AKI and can be detected in both blood and urine (18). Thus, Cys-C and NGAL are reliable early biomarkers for detecting CIAKI. In this study, there was no statistically significant difference in Scr levels between the two groups 48 h post-PCI. However, 48 h after PCI, the dapagliflozin group had significantly lower levels of Cys-C and NGAL compared to the control group. This difference in the levels of Cys-C and NGAL between the two groups at 48 h after PCI, and the 48-h elevation in scr and 1 week recovery rate were similar in both groups could be related to the sensitivity differences between Scr, Cys-C, and NGAL in the early diagnosis of AKI.

The pathogenesis of CIAKI involves complex physiological and biochemical processes (19, 20). After contrast agent administration, renal vasoconstriction reduces renal perfusion and blood flow, leading to ischemia and hypoxia in renal tissues, most pronounced in the renal medulla. This triggers oxidative stress, characterized by increased production of reactive oxygen species (ROS), which damages renal tubular cells, causing apoptosis and necrosis. Ischemia and oxidative stress also activate inflammatory responses, releasing cytokines and chemokines that further aggravate tubular injury and promote cell death. The combined effects of ischemia, oxidative stress, and inflammation contribute to CIAKI, resulting in elevated serum creatinine levels and reduced urine output post-PCI. Thus, strategies that inhibit inflammation, reduce oxidative stress, and improve renal perfusion are crucial for preventing and treating CIAKI.

In recent years, SGLT2i, particularly dapagliflozin, have emerged as a promising class of medications for managing T2DM, with notable cardiovascular and renal protective effects. Dapagliflozin works by inhibiting glucose reabsorption in the renal tubules, promoting urinary glucose excretion, and effectively controlling blood glucose levels in T2DM patients (21, 22). Beyond glucose control, dapagliflozin improves cardiac energy metabolism, reduces myocardial fibrosis, and mitigates oxidative stress and inflammation. It also lowers blood pressure, reduces body weight, increases urinary sodium excretion, and decreases kidney hyperfiltration (23). Additionally, dapagliflozin can reduce urinary protein, improve renal function in T2DM patients, and delay the progression of chronic kidney disease (24). These combined effects contribute to improved cardiovascular and renal outcomes in T2DM patients.

Although the cardiovascular and renal benefits of dapagliflozin in T2DM are well-established, its effect on CIAKI remains unclear. Some studies suggest that SGLT2i may increase the risk of AKI (25) due to several factors: (1) SGLT2i induce osmotic diuresis by increasing urinary glucose excretion, which can reduce intravascular volume and renal perfusion, especially in vulnerable patients, potentially leading to prerenal AKI in cases of dehydration, low blood pressure, chronic kidney disease, heart failure, or concurrent diuretic use (26); (2) SGLT2i decrease sodium reabsorption in the proximal tubule, raising sodium levels in the macula densa, which constricts afferent arterioles and reduces renal blood flow (27); (3) Elevated glucose concentrations in the renal tubules can alter fructose metabolism, inducing oxidative stress, uric acid production, and the release of inflammatory mediators, leading to tubular damage (28). (4) SGLT-2 inhibitors exacerbate renal medullary ischemia and hypoxia (29). These factors can cause a significant decline in eGFR and increase the risk of AKI. Consequently, some clinicians recommend discontinuing SGLT2i before PCI to minimize the risk of CIAKI.

Recent clinical data suggests that dapagliflozin does not increase the risk of AKI; in fact, it may reduce its incidence and demonstrate favorable renal safety. A study from the Nuffield Department of Population Health, published in *The Lancet*, found that SGLT2i lower AKI risk by 23% (OR = 0.77, 95% CI: 0.70–0.84) (30). Bailey et al. reported that SGLT2i reduce AKI occurrence by inhibiting oxidative stress responses (31). Additionally, the DECLARE study showed that the dapagliflozin group had a lower incidence of AKI compared to the placebo group (1.5% vs. 2.0%, HR = 0.69, 95% CI: 0.55–0.87) (32).

Clinical data show that eGFR may transiently decline during the first 1–4 weeks of SGLT2i therapy, especially in the first 2 weeks (33–35), likely due to the diuretic effect of SGLT2i, which reduces blood pressure, decreases renal blood volume, and constricts afferent arterioles. However, eGFR typically returns to baseline or normal levels after 4 weeks of treatment. Therefore, in this study, patients were required to take dapagliflozin for at least 4 weeks prior to PCI. In this study, the average duration of dapagliflozin application before PCI in the dapagliflozin group was 10.56 ± 2.62 weeks. Additionally, during the perioperative period, patients received adequate hydration to dilate renal blood vessels, increase renal blood volume, and facilitate contrast agent excretion.

This study found that the incidence of CIAKI was lower in the dapagliflozin group compared to the control group. Logistic regression analysis identified dapagliflozin as an independent protective factor against CIAKI. At 48 h post-PCI, the dapagliflozin group showed lower levels of Cys-C and NGAL than the control group. These results suggest that, with adequate hydration, dapagliflozin does not increase the risk of CIAKI. At the same time, the latest meta-analysis showed that chronic treatment (with a minimum duration of 2 weeks to 6 months) using an SGLT2 inhibitor was associated with a significantly reduced risk of CI-AKI in T2DM patients undergoing coronary procedures, compared with the control group (RR = 0.48,95%CI:0.39–0.59, *p <* 0.001) (36). Additionally, Huang et al. (37) reported that dapagliflozin may improve CIAKI by inhibiting the HIF-1α/HE4/NF-κB signaling pathway. Yet, lower HIF 1 alpha in tubular cells could simply reflect improved cortical oxygenation, as shown by O’Neill et al. (38), and HIF-mediated gene expression might be renoprotective. Therefore, for T2DM patients on long-term SGLT2i therapy, there is no need to discontinue SGLT2i before angiography, as it is safe. Clinicians need not worry about an increased risk of CIAKI from SGLT2i and can continue its use during the PCI perioperative period.

However, there is some controversy regarding the impact of short-term SGLT2i use on CIAKI. Recently, only one study has reported on the association between short-term SGLT2i use and CIAKI in T2DM patients. This study showed that the incidence of CIAKI in patients using SGLT2i for a short term (1.7 ± 1.4 days) was significantly higher than in those using SGLT2i for a long term (191.5 ± 223.3 days) (20.5% vs. 3.4%, *p* = 0.018) (39). Therefore, the impact of short-term SGLT2i use on the incidence of CIAKI requires further investigation. And for patients who are planned for angiography and intend to start SGLT2i treatment, it is advisable to delay the initiation of SGLT2i until the contrast agent is fully cleared from the body, to avoid initiating SGLT2i during the PCI perioperative period.

Currently, several clinical trials (40, 41) have shown that long-term use of SGLT2 inhibitors is beneficial for cardiovascular outcomes in T2DM patients. In this study, patients in the dapagliflozin group had better long-term outcomes compared to the control group. Considering the impact of SGLT2i on CIAKI, it may be related to the duration of SGLT2i use and considering that there was no difference in the prevention of AKI or the time of recovery from CIAKI by SGLT2i.The difference in long-term outcomes could be related to the beneficial effects of the SGLT2 inhibitor itself rather than its effects on CIAKI.

## Conclusion

Our study provides strong evidence that chronic administration of dapagliflozin significantly reduces the risk of CIAKI and improves prognosis in T2DM patients undergoing PCI. However, several limitations must be considered. First, this retrospective cohort study has a limited sample size and potential selection bias due to the absence of propensity score matching. Second, the non-randomized treatment allocation may have introduced unmeasured factors influencing the decision to prescribe dapagliflozin, which could affect the outcomes. Meanwhile, in this study, we did not measure the proteinuria/albuminuria parameters, and therefore, we were unable to observe changes in proteinuria/albuminuria or assess the impact of dapagliflozin on proteinuria/albuminuria. Third, notably, the lower LVEF in the dapagliflozin group may reflect clinicians’ preference for dapagliflozin in patients with heart failure and reduced LVEF, this selection bias may have a potential impact on the study results. Fourth, as is well known, NGAL can significantly increase 2 h after PCI, peak at 4 h, and then gradually decline (19). Our study only measured NGAL levels at 48 h post-PCI, which may not reflect the early peak levels. Additionally, the study does not assess the impact of short-term dapagliflozin use on CIAKI, and the exact mechanisms by which dapagliflozin reduces CIAKI remain unclear. Finally, since this study excluded high-risk populations for CIAKI, such as those with advanced CKD, hemodynamic instability, and emergent PCI, it cannot assess the impact of SGLT2i on the incidence of CIAKI in these high-risk groups, further research is needed to analyze the effects of SGLT2i on such high-risk populations. Therefore, further prospective studies are needed to confirm these findings and explore the underlying mechanisms.

## Data Availability

The raw data supporting the conclusions of this article will be made available by the authors, without undue reservation.
